# Alpha-Terpineol production from an engineered *Saccharomyces cerevisiae* cell factory

**DOI:** 10.1186/s12934-019-1211-0

**Published:** 2019-09-23

**Authors:** Chuanbo Zhang, Man Li, Guang-Rong Zhao, Wenyu Lu

**Affiliations:** 1 School of Chemical Engineering and Technology, Tianjin University, Tianjin, 300350 People’s Republic of China; 2 Key Laboratory of System Bioengineering (Tianjin University), Ministry of Education, Tianjin, 300350 People’s Republic of China; 3 SynBio Research Platform, Collaborative Innovation Center of Chemical Science and Engineering (Tianjin), Tianjin, 300350 People’s Republic of China

**Keywords:** Alpha-Terpineol, Monoterpene, Synthetic biology, *Saccharomyces cerevisiae*, Mevalonate pathway

## Abstract

**Background:**

Alpha-Terpineol (α-Terpineol), a C_10_ monoterpenoid alcohol, is widely used in the cosmetic and pharmaceutical industries. Construction *Saccharomyces cerevisiae* cell factories for producing monoterpenes offers a promising means to substitute chemical synthesis or phytoextraction.

**Results:**

α-Terpineol was produced by expressing the truncated α-Terpineol synthase (tVvTS) from *Vitis vinifera* in *S. cerevisiae*. The α-Terpineol titer was increased to 0.83 mg/L with overexpression of the rate-limiting genes *tHMG1*, *IDI1* and *ERG20*^*F96W-N127W*^. A GSGSGSGSGS linker was applied to fuse ERG20^F96W-N127W^ with tVvTS, and expressing the fusion protein increased the α-Terpineol production by 2.87-fold to 2.39 mg/L when compared with the parental strain. In addition, we found that farnesyl diphosphate (FPP) accumulation by down-regulation of *ERG9* expression and deletion of *LPP1* and *DPP1* did not improve α-Terpineol production. Therefore, *ERG9* was overexpressed and the α-Terpineol titer was further increased to 3.32 mg/L. The best α-Terpineol producing strain LCB08 was then used for batch and fed-batch fermentation in a 5 L bioreactor, and the production of α-Terpineol was ultimately improved to 21.88 mg/L.

**Conclusions:**

An efficient α-Terpineol production cell factory was constructed by engineering the *S. cerevisiae* mevalonate pathway, and the metabolic engineering strategies could also be applied to produce other valuable monoterpene compounds in yeast.

## Background

Terpenoids are an important class of natural products with rich chemical diversity [1]. Isopentenyl diphosphate (IPP) and dimethylallyl pyrophosphate (DMAPP) are the universal C_5_ precursors for synthesizing monoterpenoids (C_10_), sesquiterpenoids (C_15_), diterpenoids (C_20_) and triterpenoids (C_30_) among other terpenoid compounds [2]. α-Terpineol with the chemical formula C_10_H_18_0 is a monoterpenoid compound existing in plants; it was often used as perfume and repellent in the cosmetic industry and as an anticonvulsant agent in the pharmaceutical industry [3, 4]. α-Terpineol can also be used as a monomer to produce copolymers [5]. Generally, monoterpenoid is synthesized in plants via two pathways: the methyl-d-erythritol 4-phosphate (MEP) pathway and the mevalonate (MVA) pathway. The MEP pathway is located in plastid and the MVA pathway is mainly located in the cytosol in plant cells. IPP and DMAPP are all derived from the two pathways [6]. IPP and DMAPP are then condensed to geranyl diphosphate (GPP), the direct precursor of monoterpenes, by GPP synthase. Traditionally, monoterpenes are synthesized by plants in low amounts; they are mainly produced by chemical or physical extraction from natural sources such as plants [1, 3, 4]. However, this extraction is highly dependent on the acquisition of raw materials [1]. α-Terpineol production from α-pinene by homogeneous acid catalysis has also been reported, although this involved environmentally hazardous catalysts [5]. Synthetic biology provides a feasible way to discover, produce, and diversify these high-value terpenoids [7].

In recent years, with the development of synthetic biology, different microbial cell factories have been evaluated for production of monoterpene [8–10]. Among these, *S. cerevisiae* became the preferred host owing to its clear genetic background [11]. Recently, the highest geraniol titer (2 g/L) was achieved by controllable aqueous-organic two-phase fermentation [12]. In *S. cerevisiae*, IPP and DMAPP are derived via the MVA pathway and condensed to farnesyl diphosphate (FPP) by the farnesyl diphosphate synthase (ERG20). Therefore, there is no specific enzyme for GPP synthesis in *S. cerevisiae*. In *E. coli*, heterologous geranyl diphosphate synthase (GPPS) is often applied as the first enzyme to start monoterpene biosynthesis [13], while expression of heterologous GPPS in *S. cerevisiae* did not exhibit positive effects on monoterpene production [14]. It has been reported that a small number of winemaking yeast strains are able to produce trace amounts of monoterpenes [15] which implies the existence of non-specific GPPS. An *ERG20*^*K197G*^ mutation was made by Fischer and his colleagues in 2011 which enabled *S. cerevisiae* to produce 5 mg/L of geraniol [16]. Subsequently, ERG20 protein was rationally designed by Ignea and his colleagues into a GPP synthase. The engineered GPP synthase was expressed in diploid *S. cerevisiae* cells, and the resulting strain displayed a significant increase in monoterpene sabinene yield [17]. In order to improve the yield of monoterpene in yeast, the upstream MVA pathway (before *ERG20*) genes, *tHMGI*, *IDI1*, and even *ERG2*0, are frequently overexpressed [11]. *MAF1*, a negative regulator of tRNA biosynthesis that shares the same precursors as GPP, was reported to have positive effects on geraniol production when deleted in *S. cerevisiae* [18]. The downstream MVA pathway (after *ERG20*) is often weakened by *ERG9* down-regulation or *upc2.1* (transcription factor depressing ergosterol biosynthesis) overexpression [14]. It has been reported that the deletion of one *ERG9* allele in a diploid strain resulted in a drastic decrease in monoterpene cineole production [11]. However, a CEN.PK-113-5D *S. cerevisiae* strain was engineered to produce monoterpene linalool and yielded a two-fold titer increase with *ERG9* down-regulation [19]. These completely contradictory results prompted us to study the influence of the downstream MVA pathway on monoterpene synthesis in *S. cerevisiae*.

**Fig. 1:**
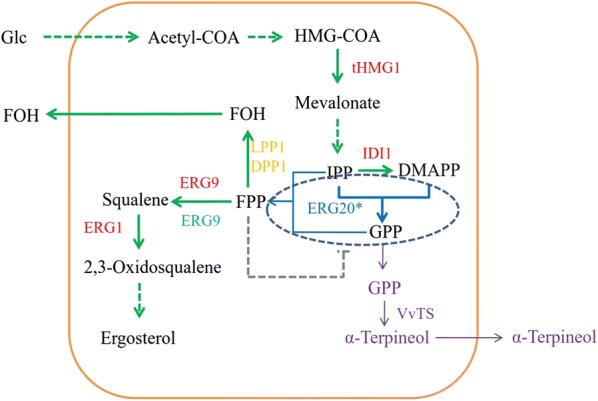
Schematic overview of α-Terpineol biosynthesis in *S. cerevisiae.* The green arrows are the endogenous pathway in *S. cerevisiae*, and the purple indicates heterogeneous genes. Blue represents protein mutation, and the gray dashed line represents supposed feedback inhibition. Genes marked in red are overexpressed, *ERG9* in cyan is weakened, and thoes in orange are deleted. *Glc*, glucose; acetyl-CoA, acetyl coenzyme A; *tHMG1*, truncated HMG-CoA reductase gene; HMG-CoA, 3-hydroxy-3-methylglutaryl-CoA; IPP, isopentenyl pyrophosphate; DMAPP, dimethylallyl pyrophosphate; *IDI1*, isopentenyl-diphosphate delta-isomerase gene; ERG20*, farnesyl diphosphate synthetase mutant gene; *ERG9*, squalene synthetase gene; FPP, farnesyl diphosphate; FOH, farnesol; *LPP1*, phosphatidate phosphatase gene *LPP1*; *DPP1*, phosphatidate phosphatase gene *DPP1*; *ERG1*, squalene monooxygenase synthase gene; GPP, geranyl diphosphate; VvTS, α-Terpineol synthase from *Vitis vinifera*

In this study, an *S. cerevisiae* cell factory was built to produce monoterpene α-Terpineol. When an ERG20^F96W-N127W^-(GS)_5_-tVvTS fusion protein was applied, in addition to the increase in α-Terpineol titer, the FOH yield was also increased which indicated a higher accumulation of FPP. Therefore, the *ERG9* gene was further overexpressed to drain the FPP pool to squalene which increased the α-Terpineol titer to 3.32 mg/L. The best α-Terpineol producing strain LCB08 was used for batch and fed-batch fermentation, and finally, 21.88 mg/L of α-Terpineol was achieved. The strategies used in this study are schematically presented in Fig. 1.

## Results

### Expression of α-Terpineol synthase in *S. cerevisiae*

**Fig. 2:**
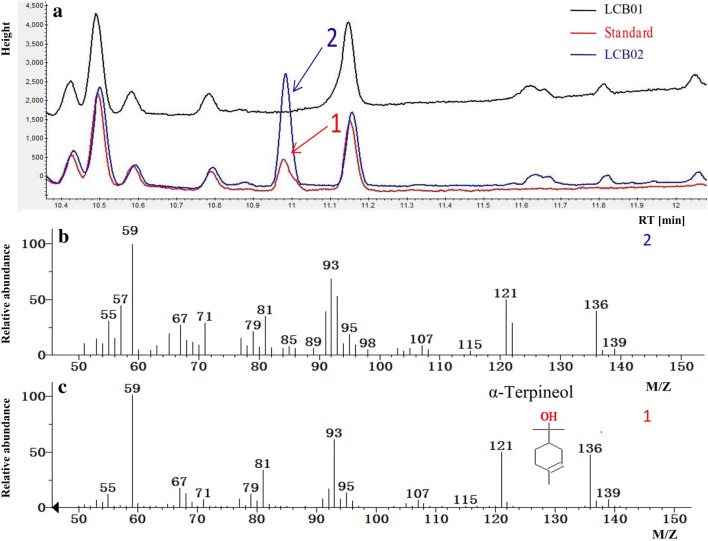
GC-MS analysis of α-Terpineol produced by engineered *S. cerevisiae* strain. **a** Chromatogram for α-Terpineol production by strain LCB02 and the control strain LCB01. **b** GC–MS spectra of the new peak (peak 2, RT = 10.98 min) produced by LCB02. **c** GC–MS spectra for the α-Terpineol standard. Peak 1 is the α-Terpineol standard; peak 2 is α-Terpineol produced by strain LCB02

*Saccharomyces cerevisiae* strain W303-1a with the co-overexpressed MVA pathway rate-limiting genes *tHMG1* and *IDI1* was selected as the original strain LCB01. The α-Terpineol synthase (VvTS) gene from *Vitis vinifera* was codon-optimized and cloned to Pxp320 with the chloroplast targeting peptide removed, according to the prediction of ChloroP (http://www.cbs.dtu.dk/services/ChloroP/) resulting in Pxp320-tVvTS. To evaluate the expression of α-Terpineol synthase, the plasmid Pxp320-tVvTS was transformed to LCB01 resulting in LCB02. A new peak at 10.98 min was detected in LCB02 but not in LCB01 and it was identified as α-Terpineol by comparing its mass spectrogram to α-Terpineol standard (Fig. 2). The titer of α-Terpineol was quantified to be 0.55 mg/L according to the standard curve.

### FPP accumulation has negative effects on α-Terpineol production

**Fig. 3:**
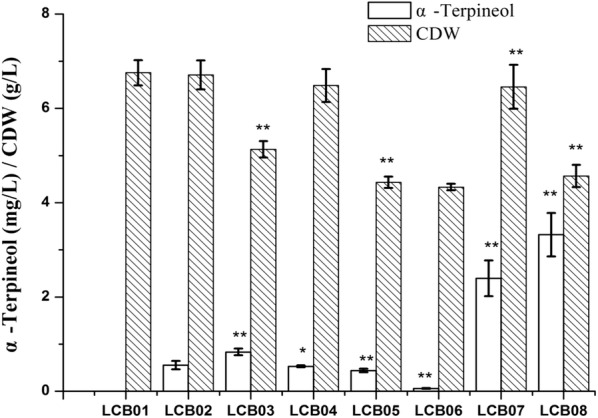
Production of α-Terpineol by different *S. cerevisia*e strains. YPD medium was used for strain cultivation, and 10% dodecane was added for two-phase fermentation. Error bars represent the standard deviation of three independent experiments. Statistically significant differences are indicated by asterisks: one asterisk indicates p < 0.05 and two asterisks indicate p < 0.001 respectively

In wild *S. cerevisiae* strains, there is no specific GPP synthase. To increase the monoterpenoid precursor GPP supply, the native FPP synthase (ERG20) was mutated to ERG20^F96W-N127W^ or ERG20^F96W-N127W-K197G^, resulting in LCB03 and LCB04, respectively. As shown in Fig. 3, the LCB03 strain harboring ERG20^F96W-N127W^ achieved a higher α-Terpineol titer (0.83 mg/L) than that in LCB04. In order to reduce the flux from FPP to squalene, the squalene synthase gene (*ERG9*) was down-regulated by replacing its native promoter with *P*_*met3*_, and the α-Terpineol titer in the engineered strain LCB05 decreased to 0.44 mg/L. Further deletion of *LPP1* and *DPP1*, which are responsible for catalyzing FPP to FOH decreased α-Terpineol production to 0.06 mg/L in LCB06.

In order to evaluate the influence of FPP content on α-Terpineol production, the yield of its downstream byproducts FOH and squalene were examined in engineered strains. As shown in Table 1, the strain LCB05 with squalene synthase weakened has the highest FOH accumulation, which indicated the highest FPP accumulation in that strain. Though the *LPP1* and *DPP1* genes were deleted in LCB06, the FOH content was still higher than in LCB03. This means other phosphatidate phosphatases were still functional in LCB06.

**Table 1:** FOH and squalene yields in engineered α-Terpineol production strains

Strains	FOH (mg/g CDW)	Squalene (mg/g CDW)
LCB01	3.41 ± 0.22	5.35 ± 0.43
LCB02	3.23 ± 0.63	5.12 ± 0.24
LCB03	3.01 ± 0.59*	4.92 ± 0.55*
LCB04	3.11 ± 0.38	4.32 ± 0.69*
LCB05	19.62 ± 2.12**	1.28 ± 0.26**
LCB06	5.76 ± 0.22**	1.72 ± 0.15*
LCB07	5.65 ± 0.17**	6.83 ± 0.48**
LCB08	0.29 ± 0.21**	10.45 ± 0.67**

“±” represents the standard deviation of three independent experiments. Statistically significant differences are indicated by asterisks: one asterisk (*) indicates p < 0.05 and two asterisks (**) indicate p < 0.001

### Fusion of ERG20^F96W-N127W^ and tVvTPS

In order to direct the exogenous monoterpene synthase to the correct subcellular compartment and rapidly deplete GPP, Erg20p^F96W-N127W^ and tVvTS were fused with a GSGSGSGSGS linker. The functional expression of the fusion protein in LCB07 increased α-Terpineol production by 300% to 2.39 mg/L compared to the parental strain LCB03. However, the FOH yield was also increased in LCB07, which was similar to that in strains LCB05 or LCB06 (Table 1). Therefore, the *ERG9* gene was overexpressed in LCB08 and the α-Terpineol titer was increased by 38.9%, to 3.32 mg/L, when compared to the parental LCB07 strain. The expression levels of target genes (*tHMG1*, *ERG20*, *IDI1*, *ERG9*) were also confirmed by RT-qPCR as shown in Additional file 1: Figure S3.

### Batch and fed-batch fermentation

**Fig. 4:**
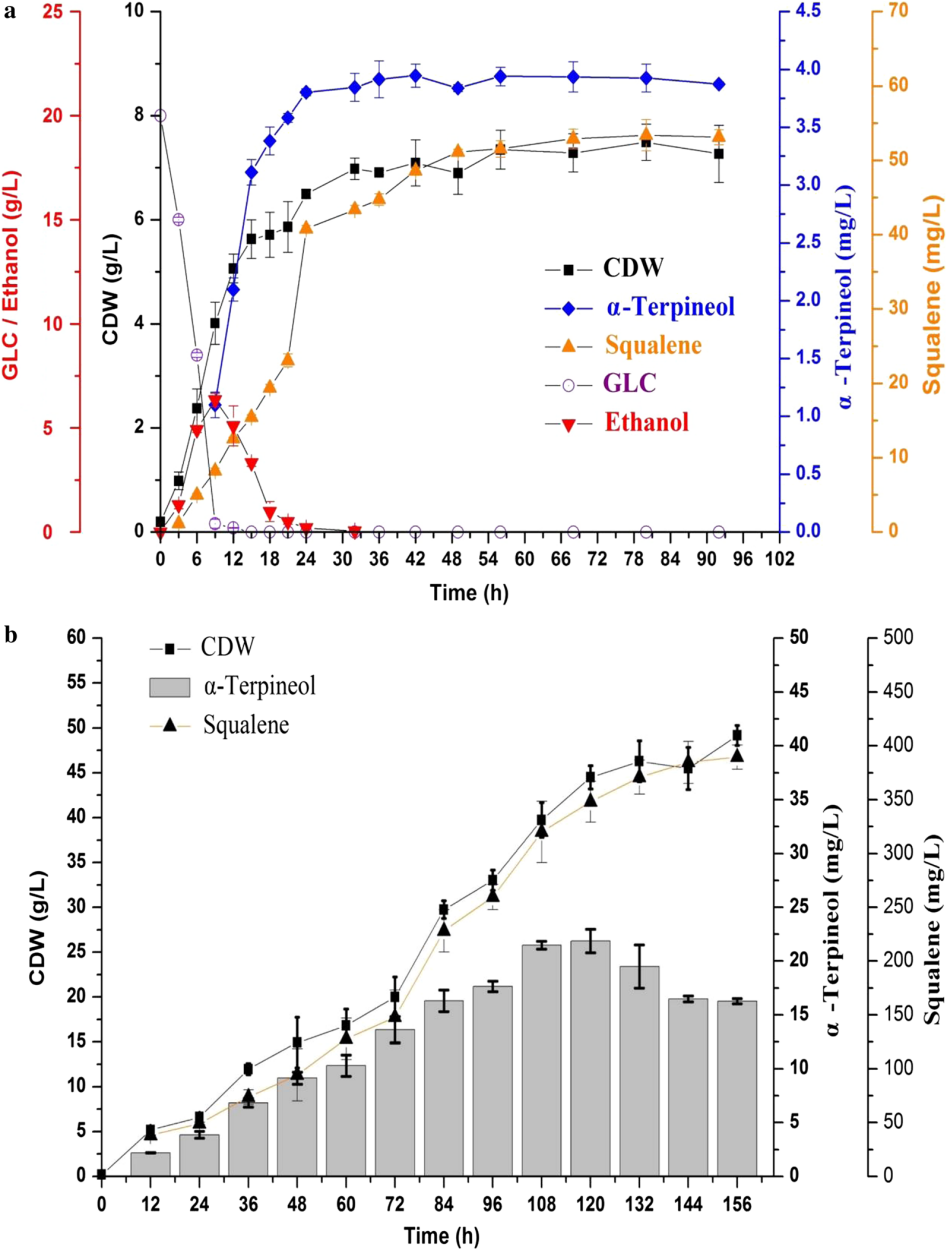
Production of α-Terpineol by batch and fed-batch cultivation LCB08 in a 5 L bioreactor. **a** Batch fermentation using LCB08 in a 5 L bioreactor. A 5 L bioreactor containing 2 L YPD medium was used for fermentation, which was conducted at 30 °C with an airflow rate of 2 vvm, and the pH was automatically maintained at 5.5. **b** Fed-batch fermentation using LCB08 in a 5 L bioreactor. The cultivation conditions were the same as for batch fermentation and feed solution was added after 24 h

The strain LCB08 was applied for batch and fed-batch fermentation. As shown in Fig. 4a, the final α-Terpineol titer in batch fermentation was 3.9 mg/L which was higher than that in shake flasks. The α-Terpineol titer was sharply increased with the depletion of ethanol along with squalene accumulation. Therefore, the feeding medium was added at 24 h. As shown in Fig. 4b, the fed-batch cultivation further increased the α-Terpineol titer to 21.88 mg/L, which was 5.6-fold higher than that in batch fermentation. The squalene content was also increased to 400 mg/L with increased biomass during the fed-batch fermentation process.

## Discussion

Monoterpenes have a simple carbon skeleton and are mainly synthesized by the MEP pathway in plants [20]. Originally, monoterpenes were mainly studied in *E. coli* and, compared to *E. coli*, only a few monoterpenes such as geraniol, linalool, limonene, and sabinene have been studied in *S. cerevisiae*. Recently, geraniol was selected as a typical monoterpene studied in *S. cerevisiae* and the highest titer (approximately 1.7 g/L) was reported by both Zhao and Jiang [20, 21]. However, both these studies were focused on the “Up-MVA” pathway (before ERG20) engineering and a further study on the “Down-MVA” pathway (after ERG20) might be helpful to exceed the highest titer. In this study, α-Terpineol was selected as the target monoterpene to study its biosynthesis in *S. cerevisiae*.

In nature, monoterpenes are mainly synthesized in plant plastids, and monoterpene synthase typically has a presequence in its N-terminus. When the full-length α-Terpineol synthase was employed, no product peak was detected (data not shown). According to software prediction, the N-terminal 28 amino acids was truncated resulting in strain LCB02, and the α-Terpineol product was detected (Fig. 2). In *S. cerevisiae*, there is no specific GPP synthase and GPP is mainly produced by farnesyl diphosphate synthase (ERG20), which is responsible for FPP synthesis [16]. However, GPP as the intermediate product of FPP synthesis is often rapidly consumed. As a result, when there is no monoterpene synthase, only trace monoterpenes are detected. In fact, the Pxp320-tVvTS plasmid was transformed into strain W303-1a, but no α-Terpineol production was detected (data not shown). The low GPP content in W303-1a was supposed to result in low α-Terpineol production which was below detection limit. In order to increase the GPP pool, the MVA pathway rate-limiting genes such as *tHMG1*, *IDI1* and *ERG20* were often overexpressed [18]. Hence, the strain LCB01 was used as the original strain for α-Terpineol production. Subsequently, researchers found that the tight binding of GPP to the FPP synthase catalytic site can be disturbed by protein engineering [16]. Native ERG20 was engineered according to both semi-rational and rational protein engineering. Three sites, 96F, 127N, and 197K, are reported to have a significant influence on GPP accumulation [16, 17]. So, we further mutated the 197K site on ERG20^F96W-N127W^ resulting in ERG20^F96W-N127W-K197G^. However, the strain LCB04 exhibited a decreased α-Terpineol titer. It seems that mutation of the three sites reduced the GPP synthetic ability of ERG20. The *ERG20* gene plays an important role in monoterpene production by *S. cerevisiae*. In a diploid *S. cerevisiae* strain engineered to produce sabinene, deletion of one *ERG20* allele increased sabinene production by 700% [17]. In another report, the authors found that irrespective of up-regulation or down-regulation *ERG20* expression, monoterpene production was decreased [14]. This complicated phenomenon might be caused by the different strain backgrounds or product categories, and even unelucidated regulatory mechanisms. So, in our study, the native *ERG20* gene was directly mutated and overexpressed using a strong promoter (LCB03). The strain LCB03 showed a growth defect but achieved higher α-Terpineol production compared to the parental LCB02 strain. It was reported that *ERG20* gene mutation results in the extension of doubling time of engineered strains when *S. cerevisiae* was engineered for monoterpene geraniol production [16]. The ERG20 catalytic site mutation was reported to lead to a low level of FPPS that could not sustain cellular metabolism since the FPP substrate plays a key role in several yeast metabolite synthetic pathways such as ergosterol, heme A, ubiquinones, and dolichols [22]. In order to enhance α-Terpineol synthase catalytic efficiency, an artificial fusion protein was constructed according to Ignea and co-workers, and the effect was consistent with other monoterpene synthases and Erg20^F96W-N127W^ artificial fusion protein [17]. Cell growth was also found to be increased in strain LCB07.

Sterols, essential membrane components of *S. cerevisiae*, are synthesized from FPP by squalene synthase (ERG9) and were often considered to be the competing branch for terpene production [23, 24]. Therefore, *ERG9* was often down-regulated for sesquiterpene synthesis, which was reported to have positive effects on its production [25–27]. However, monoterpene production via *ERG9* downregulation showed different results. In our study, when the native promoter of *ERG9* was replaced by *P*_*met3*_, α-Terpineol production was decreased drastically. Our results are consistent with those of Ignea in engineering *S. cerevisiae* for cineole production [11] but completely contradict the results of Amiri in engineering *S. cerevisiae* for linalool production [19]. These results may be due to balance point variation between the GPP pool, FPP pool and squalene pool. In our study, FPP pool accumulation caused FPP product inhibition, thus lowering the availability of GPP. It has been reported that the extracellular FOH level can reflect the intracellular FPP content [13]. As shown in Table 1, the *ERG9* downregulation strain (LCB05) had the highest FOH production. The further deletion of *DPP1* and *LPP1* confirmed our deduction, and the α-Terpineol titer was decreased with intracellular FPP accumulation (Table 1). In the case of linalool production, the FPP pool might not be sufficient for causing product inhibition. In order to relieve potential product inhibition by FPP, we selected the strain LCB07 to overexpress *ERG9*, resulting in strain LCB08. Interestingly, the titer of α-Terpineol was increased by 38.9% to 3.32 mg/L compared to LCB07, and FOH production was less than in other strains constructed in this study. When the LCB08 strain was used for batch and fed-batch fermentation (Fig. 4), squalene accumulation accompanied Terpineol production. It has been reported that squalene accumulation in *S. cerevisiae* down-regulated ethanol production and post-squalene biosynthetic pathways [28]. The comprehensive effects of squalene accumulation may also contribute to α-Terpineol or other monoterpene production.

## Materials and methods

### Strains, media, and plasmids

*Saccharomyces cerevisiae* W303-1a was used as the original strain kept in our lab [29]. Pxp218 and Pxp320 plasmids were obtained from ATCC. VvTS (GenBank: NM_001281287) from *Vitis vinifera* was synthesized by GENEWIZ (Suzhou, China) with codon optimization for *S. cerevisiae*. *E. coli* DH-5α was used for plasmid construction and was cultivated in LB medium (yeast extract, 5 g/L; tryptone, 10 g/L, NaCl, 10 g/L) supplemented with 100 mg/L ampicillin at 37 °C. SD medium (20 g/L of glucose, 1.7 g/L of yeast nitrogen base, 5 g/L of (NH_4_)_2_SO_4_, 2 g/L of synthetic complete amino acid drop-out medium) was used for *S. cerevisiae* growth and selection with suitable nutritional deficiencies.

The chloroplast targeting peptide of monoterpene synthases VvTS was predicted by ChloroP (http://www.cbs.dtu.dk/services/ChloroP/) and PSORT (http://wolfpsort.org/) programs. The VvTS was truncated 28 amino acids from the N-terminus with primers *Spe*I-Ts-28F/*Xho*I-Ts-R based on prediction and cloned into the *Spe*I and *Xho*I sites of Pxp320, resulting in Pxp320-tVvTS.

ERG20 was mutated by fusion PCR with primers (Erg20-*Spe*I-F/Erg20-*Xho*I-R, N96W-F/N96W-R, F127W-F/F127W-R, K197G-F/K197G-R) listed in Additional file 1: Table S1 and was cloned into pEASY-Blunt (TransGen Biotech, Beijing, China).

### Engineering *S. cerevisiae* for monoterpene production

Strains constructed in this study are listed in Table 2. Promoters and terminators used for gene expression were amplified from *S. cerevisiae* W303-1a genome. *P*_*pgk1*_-*tHMG1*-*T*_*pgk1*_, *P*_*TDH3*_-*IDI1*-*T*_*ADH1*_, *P*_*pgk1*_-*ERG20*^*(F96W*-*N127W)*^-*(GS)*_*5*_-*28TS*-*T*_*ADH1*_, and *P*_*TDH3*_-*ERG9*-*T*_*ADH1*_ expression cassettes were constructed by fusion PCR according to our previously reported methods [30, 31]. *S. cerevisiae* transformations were performed according to [32]. The termini of the gene expression cassettes had at least 300 bp of homologous arms with *S. cerevisiae* genome insertion sites and overlaps between gene expression cassettes were at least 40 bp. The detailed construction process is illustrated in Additional file 1: Figure S1 and primers used in this study were presented in Additional file 1: Table S1. The genome DNA of engineered strains was extracted according to standard methods and was then used for PCR verification with two primers which were designed to bind the gene expression cassette and the genome integration site, respectively. The gene expression cassette was PCR cloned and then sequenced to select an appropriate clone. An electrophoretogram was provided in Additional file 1: Figure S2 and primers used are shown in Additional file 1: Table S2. The copy number of target genes were determined by qPCR according to our previous methods [29] as shown in Additional file 1: Figure S3.

**Table 2:** Strains used in this study

Strains	Description	Parental strain
W303-1a	*MATa*; *leu2*-*3*,*112*; *trp1*-*1*; *can1*-*100*; *ura3*-*1*; *ade2*-*1*; *his3*-*11*,*15*	–
LCB01	*δ::P*_*pgk1*_-*tHMG1*-*T*_*pgk1*_-*P*_*TDH3*_-*IDI1*-*T*_*ADH1*_	W303-1a
LCB02	*δ::P*_*pgk1*_-*tHMG1*-*T*_*pgk1*_-*P*_*TDH3*_-*IDI1*-*T*_*ADH1*_; Pxp320-tVvTS	LCB01
LCB03	*δ::P*_*pgk1*_-*tHMG1*-*T*_*pgk1*_-*P*_*TDH3*_-*IDI1*-*T*_*ADH1*_; *erg20*::P_pgk1_-*ERG20*^(F96W-N127W)^; Pxp320-tVvTS	LCB02
LCB04	*δ::P*_*pgk1*_-*tHMG1*-*T*_*pgk1*_-*P*_*TDH3*_-*IDI1*-*T*_*ADH1*_; *erg20*::P_pgk1_-*ERG20*^*(F96W*-*N127W*-*K197G)*^; Pxp320-tVvTS	LCB02
LCB05	*δ::P*_*pgk1*_-*tHMG1*-*T*_*pgk1*_-*P*_*TDH3*_-*IDI1*-*T*_*ADH1*_; *erg20*::P_pgk1_-*ERG20*^(F96W-N127W)^; *Pmet3*-*erg9*; Pxp320-tVvTS	LCB03
LCB06	*δ::P*_*pgk1*_-*tHMG1*-*T*_*pgk1*_-*P*_*TDH3*_-*IDI1*-*T*_*ADH1*_; *erg20*::P_pgk1_-*ERG20*^(F96W-N127W)^; *Pmet3*-*erg9*; *LPP1*:: loxp; *DPP1*::loxp; Pxp320-tVvTS	LCB05
LCB07	*δ::P*_*pgk1*_-*tHMG1*-*T*_*pgk1*_-*P*_*TDH3*_-*IDI1*-*T*_*ADH1*_; *erg20*::P_pgk1_-*ERG20*^(F96W-N127W)^; *rDNA*::*P*_*pgk1*_-*ERG20*^*(*F96W-N127W*)*^-*(GS)*_*5*_-tVvTS-*T*_*ADH1*_; Pxp320-tVvTS	LCB03
LCB08	*δ::P*_*pgk1*_-*tHMG1*-*T*_*pgk1*_-*P*_*TDH3*_-*IDI1*-*T*_*ADH1*_; *erg20*::P_pgk1_-*ERG20*^(F96W-N127W)^; *rDNA*::*P*_*pgk1*_-*ERG20*^*(*F96W-N127W*)*^-*(GS)*_*5*_-tVvTS-*T*_*ADH1*_; *HO::P*_*TDH3*_-*ERG9*-*T*_*ADH1*_;Pxp320-tVvTS	LCB07

### Monoterpene production in shake-flask cultures

Engineered *S. cerevisiae* strains were cultured in SD medium with suitable nutrition deficiency for 24 h, and the culture was then inoculated into Erlenmeyer flasks containing 30 mL YPD medium and 2 mL dodecane. The culture conditions were 220 rpm, 30 °C for 4 days. Parallel fermentation experiments were performed in triplicate. Statistical data analyses were determined by Student’s t-test, and p < 0.05 was considered significant.

### Batch and fed-batch cultivations

The strain LCB08 was used for batch and fed-batch fermentation. A single colony obtained from a plate was inoculated into 5 mL YPD medium for 24 h, and then the culture was transferred into a 500 mL flask containing 100 mL of medium and grown for 16 h. Then, the culture was used as the seed for a 5-L fermenter (ShangHai BaoXing Bio-Engineering, China) containing 2 L YPD medium. Fermentation was carried out at 30 °C, 600 rpm with an air flow rate of 2 L/min. The pH was controlled at 5 with 2 M NaOH and 2.5 M H_2_SO_4_ automatically. 10% (v/v) dodecane was added after 10 h of incubation. For fed-batch fermentation, the initial culture conditions (before 24 h) were the same as for the 5 L batch fermentation. After 24 h, the feed solution was used as acid, and ammonia was used as alkali to maintain pH around 5.0. One liter of feed solution contained: 500 g glucose, 9 g KH_2_PO_4_, 3.5 g K_2_SO_4_, 0.28 g Na_2_SO_4_, 5.12 g MgSO_4_·7 H_2_O, 10 mL microelement stock solution (1 L containing: 10.2 g, ZnSO_4_·7H_2_O, 15 g, EDTANa_2_·2H_2_O, 5.12 g FeSO_4_·7H_2_O, 0.5 g CuSO_4_, 0.5 g MnCl_2_·4H_2_O, 0.86 g CoCl_2_·6H_2_O, 3.84 g CaCl_2_·2H_2_O, 0.56 g Na_2_MoO_4_·2H_2_O) and 12 mL vitamin stock solution (1 L containing: 25 g inositol, 0.05 g biotin, 1 g niacin, 1 g calcium pantothenate, 1 g thiamine HCl, 1 g pyridoxol HCl, 0.2 g *p*-aminobenzoic acid). In all, 1 L feed solution was supplied from 24 h until the end.

### Metabolites analysis

Glucose and ethanol levels were measured by Bioanalyzer (SBA-40C, Shandong Academy of Sciences, China) following the manufacturer’s instructions. OD600 was measured with a spectrophotometer (Oppler, 752N, China). CDW (g/L) = OD_600_ × 0.33. Squalene was extracted and quantified according to [29].

### RNA extraction and quantitative real-time PCR (RT-qPCR)

Cells (1 × 10^7^) at early-log growth phase were collected for RNA extraction using the RNeasy Minikit (Tiangen, China) according to the manufacturer’s instruction. RNase-free DNase I was used for elimination of genomic DNA during the extraction process. Then, cDNA was synthesized using Maxima H Minus First Strand cDNA Synthesis Kit (Fermentas, USA) according to the manufacturer’s recommendations. Actin gene *ACT1* was used as an internal reference gene and oligonucleotides used for qPCR are listed in Additional file 1: Table S3. Real-time PCR was conducted according to our previous studies [33, 34]. The results were normalized using the *ACT1* gene as the reference gene and presented as ratios of gene expression between the engineered strains and the control strains [35] (Additional file 1: Figure S4).

### α-Terpineol extraction, identification, and quantification

Five milliliters of medium (the mixture of dodecane and medium) was sampled and centrifuged at 12,000*g* for 10 min, and then 50 μL of the dodecane layer was transferred to another tube at − 20 °C for further analysis. α-Terpineol was identified by GC–MS (Agilent Technologies 7890 A GC system equipped with a 5975 C insert 143 XL EI/CI MSD Detector) with a DB-WAX column (30 m × 0.32 mm × 0.25 μm). The amount of α-Terpineol was determined using linear calibration curves. For GC analysis, 1 μL of dodecane sample was injected with a split ratio of 20:1 and nitrogen was used as the carrier gas with a flow rate of 1 mL/min. The injector and detector temperature were maintained at 250 °C. The oven temperature was as follows: 80°C for 1 min and sequentially increased at the rate of 10 °C/min to 180 °C and 30 °C/min to 250 °C.

## Supplementary information

**Additional file 1**: **Figure S1.** Expression cassette construction and insertion. **Figure S2.** Genetically engineered strain verification. **Figure S3.** The copy number of target genes determined via qPCR. **Figure S4.** RT-qPCR analysis of engineered target genes. **Table S1.** Primers used for expression cassette construction in this study. **Table S2.** Primers used for strain verification. **Table S3.** Primers used for quantitative real-time PCR. **Table S4.** Codon-optimized VvTS sequence for *S. cerevisiae*.

## Data Availability

All data generated or analyzed in this study are included in this published article and its Additional file.
