# Supplementary material for: Alpha-Terpineol production from an engineered Saccharomyces cerevisiae cell factory
Source: Microb Cell Fact. 2019 Sep 23;18:160. doi: 10.1186/s12934-019-1211-0 (PMC6757357; doi:10.1186/s12934-019-1211-0)
Supplement: Supplementary file 1 — Additional file 1: Figure S1. Expression cassette construction and insertion. Figure S2. Genetically engineered strain verification. Figure S3. The copy number of target genes determined via qPCR. Figure S4. RT-qPCR analysis of engineered target genes. Table S1. Primers used for expression cassette construction in this study. Table S2. Primers used for strain verification. Table S3. Primers used for quantitative real-time PCR. Table S4. Codon-optimized VvTS sequence for S. cerevisiae. [file 12934_2019_1211_MOESM1_ESM.doc]

Additional file

**Alpha-Terpineol production from an engineered *Saccharomyces cerevisiae* cell factory**

Chuanbo Zhang^a^, Man Li^a^, Guang-Rong Zhao^a,b,c^, Wenyu Lu^a.b,c^*

^a^ School of Chemical Engineering and Technology, Tianjin University, Tianjin 300350, PR China

^b^ Key Laboratory of System Bioengineering (Tianjin University), Ministry of Education, Tianjin 300350, PR China

^c^ SynBio Research Platform, Collaborative Innovation Center of Chemical Science and Engineering (Tianjin), Tianjin 300350, PR China

* Correspondence to: W. Y. Lu, School of Chemical Engineering and Technology, Tianjin University, Tianjin 300350, PR China. Tel: +86-22-85356523, Fax: +86-22-27400973. E-mail address: wenyulu@tju.edu.cn


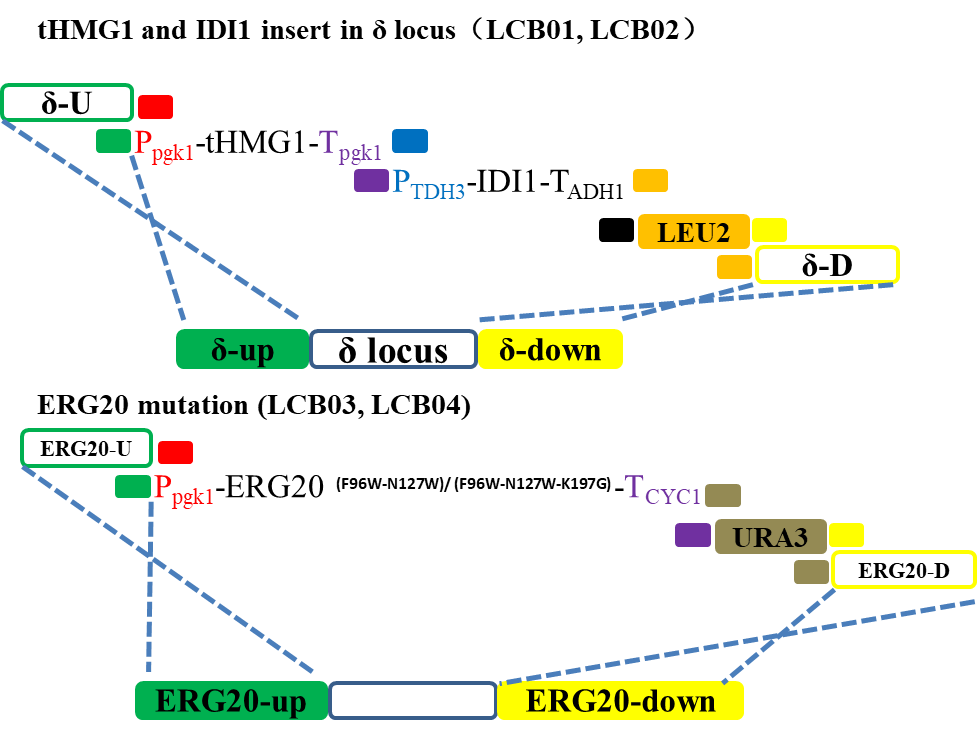


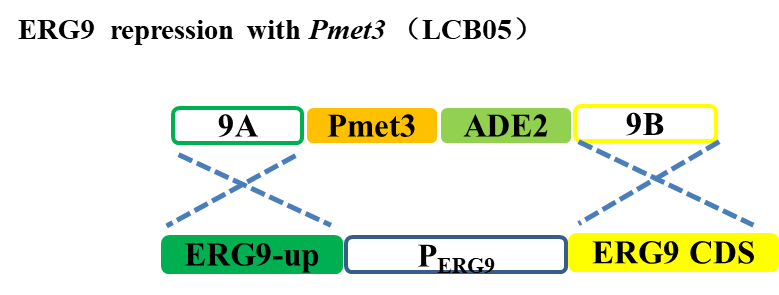


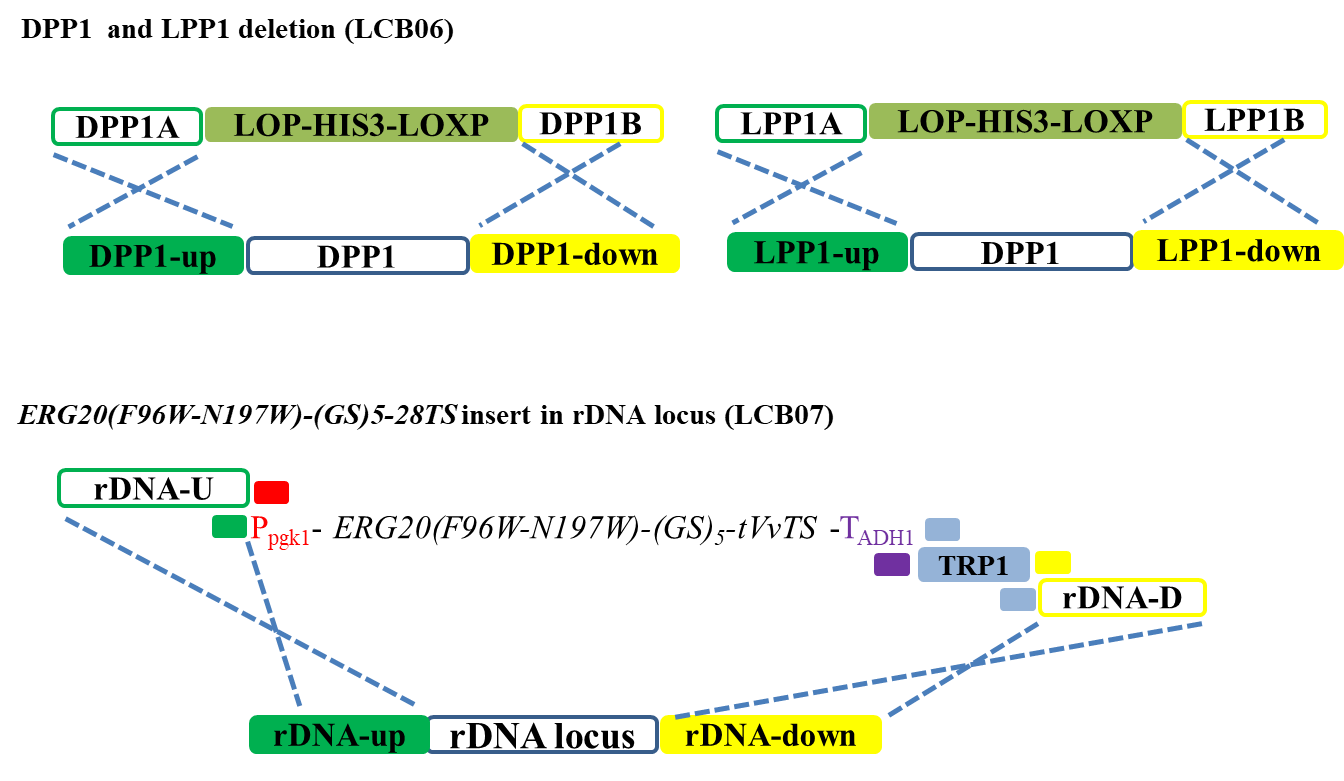


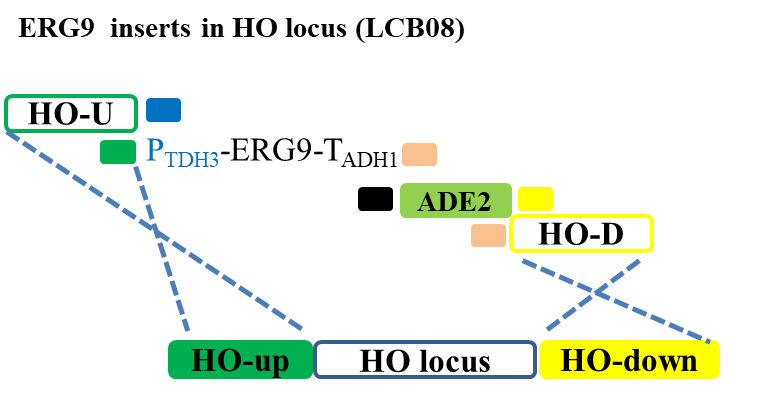


**Figure S1** Expression cassette construction and insertion


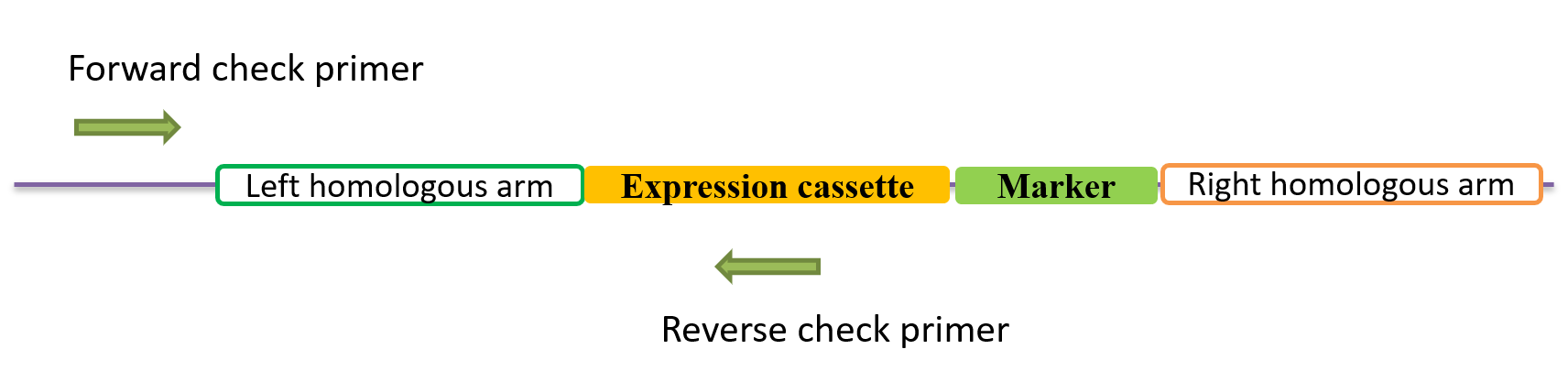


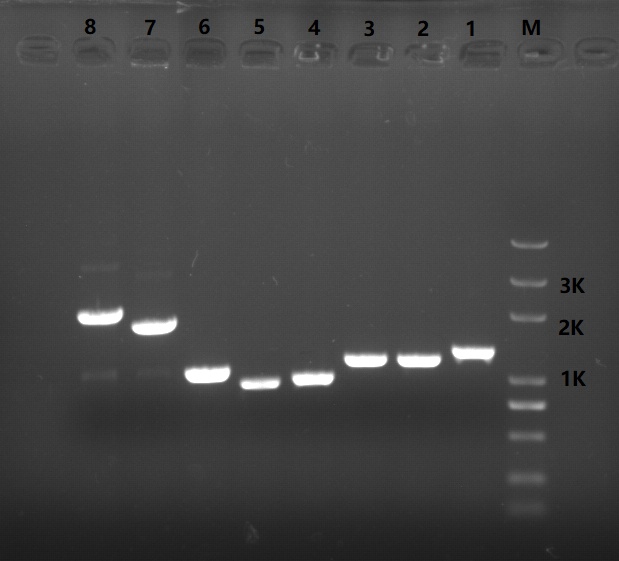


**Figure S2.** Genetically engineered strain verification. M, marker; 1, Strain LCB01 verification; 2, Strain LCB03 verification; 3, Strain LCB04 verification; 4, Strain LCB05 verification; 5, Strain LCB06 verification (*LPP1* deletion); 6, Strain LCB06 verification (*DPP1* deletion); 7, Strain LCB07 verification; 8, Strain LCB08 verification.


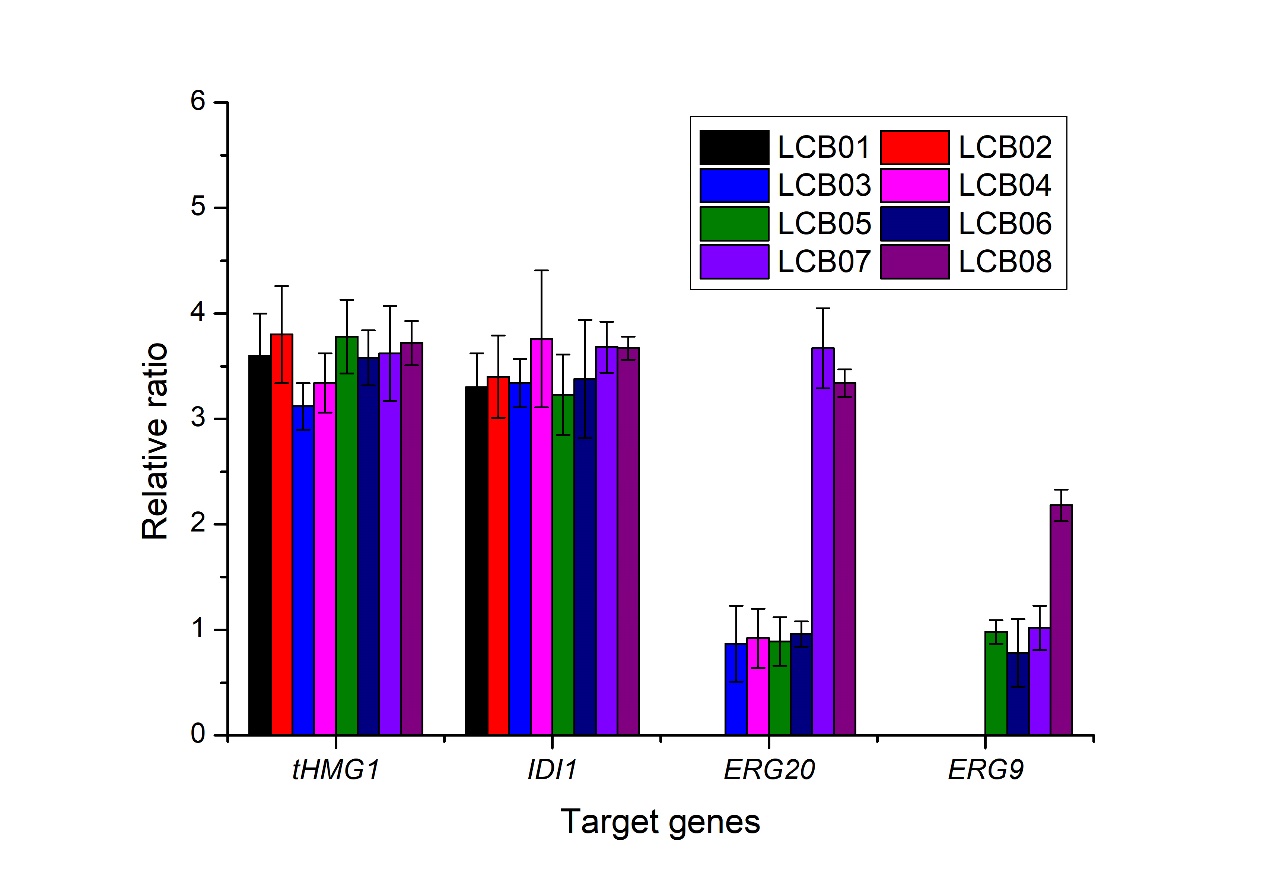


**Figure S3.** The copy number of target genes determined via qPCR. Open reading frames of *tHMG1*, *ERG20*, *IDI1*, *ERG9* were ampliﬁed and quantiﬁed to draw standard curves, respectively. The house-keeping gene *ARG4* (ampliﬁed via *ARG4*-F: TCAATGACACCCTCTTTCCAG and *ARG4*-R: AATGCCGTAAGGATGACCAG) was selected as a reference gene. Error bars represent the standard deviation of biological triplicate. qPCR was peformed according to our previous methods [1].

[1] Zhao F, Bai P, Liu T, Li D, Zhang X, Lu W, Yuan Y. Optimization of a cytochrome P450 oxidation system for enhancing protopanaxadiol production in *Saccharomyces cerevisiae*. Biotechnol Bioeng. 2016;113:1787-1795.


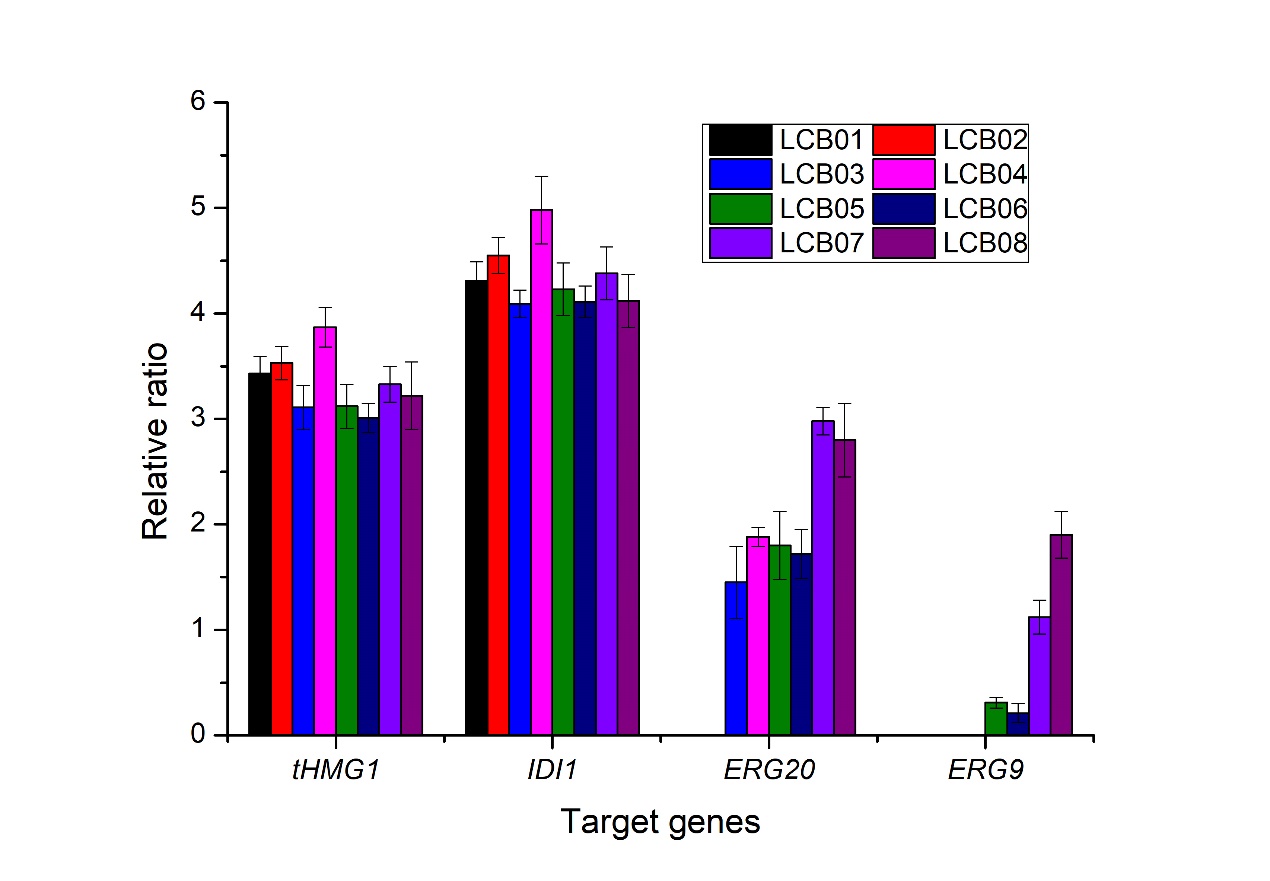


**Figure S4**. RT-qPCR analysis of engineered target genes. Expression of the MVA pathway genes, *tHMG1*, *IDI1*, *ERG20* and *ERG9*, were increased in engineered strains after the overexpression. The results are presented as the relative gene expression ratio of target genes in each strain with respect to that of the control strain (W303-1a). Error bars represent the standard deviation of biological triplicate.

**Table S1** Primers used for expression cassette construction in this study

| **Name** | **Primer sequence (5-3)** |
| --- | --- |
| ***tHMGI* and *IDI1* gene expression cassettes construction** | |
| δ2-F | GCTTCGGTTACTTCTAAGGAAGTCCACACAAATCAAGATCCGTTAG |
| PGK1-δ2-R | GTTATAATATCTGTGCGTCTTGAGTTGAAGTCAGGAATCTAAAATATTGGAAAGTCATTAGGTGAGGTT |
| δ2-PGK1-F | TATGTCAGACCACCACCAATGTTAACCTCACCTAATGACTTTCCAATATTTTAGATTCCTGACTTCAAC |
| tHMG1-PGK1-R | ATTGGTTAAAACTGGCATTGTTTTATATTTGTTGTAAAAAGTAG |
| PGK1-tHMG1-F | CAACAAATATAAAACAATGCCAGTTTTAACCAATAAAACAGTC |
| PGK1T-tHMG1-R | CTATCGATTTCAATTCAATTCAATTTAGGATTTAATGCAGGTGAC |
| tHMG1- PGK1T-F | GTCACCTGCATTAAATCCTAAATTGAATTGAATTGAAATCGATAG |
| TDH3-PGK1T-R | TAACATTCAACGCTAGTATCACTATACTGGATCTAAAGAGTACAATAG |
| PGKT-TDH3-F | CTCTTTAGATCCAGTATAGTGATACTAGCGTTGAATGTTAGCG |
| IDI-TDH3-R | CTATTGTTGTCGGCAGTCATTTTGTTTGTTTATGTGTG |
| TDH3-IDI-F | CACACATAAACAAACAAAATGACTGCCGACAACAATAG |
| ADHI-IDI-R | ATCATAAATCATAAGAAATTCGCTTATAGCATTCTATGAATTTGCC |
| IDI-ADH1-F | GGCAAATTCATAGAATGCTATAAGCGAATTTCTTATGATTTATGAT |
| LEU2-ADH1-R | CATGAACAAGGAAGTACAGGACAGATCATGATACATAAAAGCGATATAAC |
| ADH1-LEU2-F | GTTATATCGCTTTTATGTATCATGATCTGTCCTGTACTTCCTTGTTCATGTG |
| δ1-LEU2-R | GATAGTTGATTTCTATTCCAACAGGAAATGGTTCAAGAAGGTATTGAC |
| LEU2-δ1-F | GTCAATACCTTCTTGAACCATTTCCTGTTGGAATAGAAATCAACTATC |
| δ1-R | CACAGGCGCTACCATGAGAATTG |
| ***ERG20*(F96W-N127W) and *ERG20*(F96W-N127W-K197G) gene expression cassettes construction** | |
| Erg20-SpeI-F | CTCAAAACTAGTACGTAGAAATGGCTTCAG |
| Erg20-XhoI-R | TTATCGACTCGAGTTAGTTCTATTTGCTTC |
| F96W-F | GGCTTACTGGTTGGTCGCC |
| F96W-R | CGGCGACCAACCAGTAAGC |
| N127W-F | CCATCTGGGACGCATTCATG |
| N127W-R | CATGAATGCGTCCCAGATGG |
| K197G-F | CATAGTTACTTTCGGTACTGCTTACTATTC |
| K197G-R | GAATAGTAAGCAGTACCGAAAGTAACTATG |
| ERGI-F | AAGTGACAAGCAATATCATTGAGCATTC |
| pXP-ERGI-R | CTCGAAGGCTTTAATTTGCGGCCCATGAATGCGTCATTGATG |
| ERGI-pXP-F | CATCAATGACGCATTCATGGGCCGCAAATTAAAGCCTTCGAG |
| ERGII-pXP- R | GTTCTGGGGTACCGAAGCAGTGAATTCGAGCTCGGTACCCG |
| pXP-ERGII-F | CGGGTACCGAGCTCGAATTCACTGCTTCGGTACCCCAGAAC |
| ERGII-R | CCGTAATTTCCGATCACGCATTTCTTC |
| **Plasmids construction** | |
| SpeI-Ts-F | CTCGCGAATGCAaCTAGtTATGGC |
| XhoI-Ts-R | GGCtCGaGATCCGATTTATTC |
| SpeI-Ts-28F | GCACTAGTATGGCTTCACCAAGAG |
| Erg20-SpeI-F | CTCAAAACTAGTACGTAGAAATGGCTTCAG |
| Erg20-XhoI-R | TTATCGACTCGAGTTAGTTCTATTTGCTTC |
| ***ERG9* expression cassettes construction** | |
| 9A-F | AGTGCAGCTCAGAGCCCCCAGC |
| ade2-9A-R | CACGATACGGCGTTATGTGTGTGTGTGATATGTG |
| A-ADE2-F | CATATCACACACACACATAACGCCGTATCGTGATTAACG |
| Met3-ADE2-R | CTTATACCACTACGTACATATCGTTGCGCTATCCTCGGTTCTGCATTG |
| ade2-met3-F | CAATGCAGAACCGAGGATAGCGCAACGATATGTACGTAGTGGTATAAG |
| 9B-Met3-R | GCCAATTGTAATAGCTTTCCCATGTTAATTATACTTTATTCTTG |
| Met3-9B-F | CAAGAATAAAGTATAATTAACATGGGAAAGCTATTACAATTGGC |
| 9B-R | ACACGTCGTAGTCGTGGACGGTTTGCAAC |
| HOL-F | TCCCAGGCGTAGAACAGTTTATCAG |
| TDH3-HOL-R | TCAACGCTAGTATGATCCAAGCTATCTACTGAG |
| HOL-TDH3-F | AGATAGCTTGGATCATACTAGCGTTGAATGTTAG |
| ERG9-TDH3-R | ATAGCTTTCCCATTTTGTTTGTTTATGTGTGTTTATTCG |
| TDH3-ERG9-F | CACATAAACAAACAAAATGGGAAAGCTATTACAATTGGC |
| ADH1-ERG9-R | CATAAATCATAAGAAATTCGCTCACGCTCTGTGTAAAGTGTAT |
| ERG9-ADH1-F | ATACACTTTACACAGAGCGTGAGCGAATTTCTTATGATTTATG |
| ADE2-ADH1-R | ACGATACGGCGTTAAGATCATGATACATAAAAGC |
| ADH1-ADE2-F | GTATCATGATCTTAACGCCGTATCGTGATTAAC |
| HOR-ADE2-R | CTGACATACCAAGACGCTATCCTCGGTTCTGCATTG |
| ADE2-HOR-F | ACCGAGGATAGCGTCTTGGTATGTCAGCTACTG |
| HOR-R | CACTTCACGTGCTTCTGGTACATACTTG |
| ***LPP1* and *DPP1* deletion cassettes construction** | |
| LPPA-F | CCAATCATGGTTTCATGGTCACTGGTAAG |
| LPPA-His3-F | CTAAGGAAACTCGTCATATTCGTCGACTCTAGAGGATC |
| His3-LPPA-R | GATCCTCTAGAGTCGACGAATATGACGAGTTTCCTTAG |
| His3-LPPB-F | CGAATTCATCACGTGCTATAAGCTATACTACTTTCAGTAC |
| LPPB-His3-R | GTACTGAAAGTAGTATAGCTTATAGCACGTGATGAATTCG |
| LPPB-R | CTGTAAGTGTTACTAAACACTAACCGGTGAAG |
| DPP1A-F | GTCCGATAAACACAGTTGTGATCTGTCTTG |
| DPP1A-HIS-F | GCAAAGAATCAGAATTAAATCATAGTCGACTCTAGAGGATCC |
| HIS-DPP1A-R | GGATCCTCTAGAGTCGACTATGATTTAATTCTGATTCTTTGC |
| HIS-DPP1B-F | GAATTCATCACGTGCTACAGTGTATTACCAACAACCG |
| DPP1B-HIS-R | CGGTTGTTGGTAATACACTGTAGCACGTGATGAATTC |
| DPP1B-R | CGGGATCCTCTGATGGGTGACTG |
| ***ERG20*(F96W-N197W)-(GS)_5_-*TS* gene expression cassettes construction** | |
| rDNA2-F | CCGGGGCACCTGTCACTTTGGAAAAAAAATATACGCTAAGA |
| PGK1-rDNA2-R | TGGTGGAACCTGATTAGAGGAAATATTTTAGATTCCTGACTTCAACTC |
| rDNA2-PGK1-F | GAGTTGAAGTCAGGAATCTAAAATATTTCCTCTAATCAGGTTCCAC |
| GS5-ERG20- R | AGAACCAGAACCAGAACCAGAACCAGAACCTTTGCTTCTCTTGTAAAC |
| GS5-28TS-F | GGTTCTGGTTCTGGTTCTGGTTCTGGTTCTATGGCTTCACCAAGAGGTAT |
| ADH1-28TS-R | CATAAATCATAAGAAATTCGCTTATTCAAAAGACAATGATGGGATTG |
| 28TS-ADH1-F | CAATCCCATCATTGTCTTTTGAATAAGCGAATTTCTTATGATTTATG |
| TRP1-ADH1-R | GACCTGCAGGCATGCAAGATCATGATACATAAAAGCG |
| ADH1-TRP1-F | TATGTATCATGATCTTGCATGCCTGCAGGTCGAC |
| rDNA1- TRP1- R | TGCTACTCTCATACGTGATGAATTCGAGCTC |
| TRP1-rDNA1-F | GAATTCATCACGTATGAGAGTAGCAAACGTAAG |
| rDNA1-R | GCGGAAAATACGGAAACGCGCGGGAACATACAA |

**Table S2** Primers used for engineered strain verification

| **Strain** | **Name** | **Primer sequence (5-3)** |
| --- | --- | --- |
| LCB01 | Check-δ-F | CATGGTAGCGCCTGTG |
|  | Check-tHMG1-R | AAACTTTTGACTTTCGATC |
| LCB03 | Check-ERG20-F | CTTCGACATGATCACGTAAAACAACAAC |
|  | Check-ERG20-R | TTGTCCATCATATCATCGGCG |
| LCB04 | Check-ERG20-F | CTTCGACATGATCACGTAAAACAACAAC |
|  | Check-ERG20-R | TTGTCCATCATATCATCGGCG |
| LCB05 | Check-MET3-F | CATTCTAGCGGTCCCTGTCGT |
|  | Check-ERG9-R | GGCCAGAAGGATCTACCATCG |
| LCB06 | Check-LPP1-F | TGAAAACTTCACAGGGAGAAATAG |
|  | Check-HIS3-R | TTCTGCTCTGTCATCTTTGCC |
|  | Check-DPP1-F | CGAAAAGTTTGACCGTTTAG |
| LCB07 | Check-rDNA-F | TATGTTCCCGCGCGTTTCCGT |
|  | Check-ERG20-R | TTGTCCATCATATCATCGGCG |
| LCB08 | Check-HO-F | CCCGCGTCATAAATGTCACAC |
|  | Check-ERG9-R | GGCCAGAAGGATCTACCATCG |

**Table S3** Primers used for quantitative real-time PCR

| **Name** | **Length (bp)** | **Tm (°C)** | **GC content (%)** | **Primer sequence (5-3)** |
| --- | --- | --- | --- | --- |
| ACT1-F | 18 | 59 | 56 | TCCGTCTGGATTGGTGGT |
| ACT1-R | 22 | 59 | 41 | TGAGATCCACATTTGTTGGAAG |
| tHMG1-F | 20 | 59 | 50 | TGCCATCCATCGAAGTAGGT |
| tHMG1-R | 20 | 60 | 50 | CATGGCACCTTGTGGTTCTA |
| IDI1-F | 23 | 59 | 39 | TTTCCCTGATCTTTGGACTAACA |
| IDI1-R | 21 | 59 | 43 | CCCTTCAAACCTAATTCGTCA |
| ERG20-F | 20 | 60 | 50 | CCGGTATCACGGATGAAAAG |
| ERG20-R | 21 | 59 | 43 | TTCACCCAATGGAATCAAGAC |
| ERG9-F | 20 | 60 | 55 | GGCCCAAGGAAATCTGGTCA |
| ERG9-R | 20 | 60 | 55 | TGGATTGCTCGTGGATACCG |

Yuan, J., Ching, C.-B. Mitochondrial acetyl-CoA utilization pathway for terpenoid productions. Metab. Eng. 2016; 38: 303-309.

**Table S4** Codon-optimized VvTS sequence for *S. cerevisiae*

| **ATGGCTTTGTCTATGTTGTCTTCAATCCCAAATTTGATCACTCATACAAGATTGCCAATCATTATTAAATCTTCATCTTGTAAA**GCTTCACCAAGAGGTATTAAAGTTAAGATCGGTAATTCTAACTGTGAAGAAATCATCGTTAGAAGAACTGCAAACTACCATCCAACAATCTGGGATTACGATTACGTTCAATCATTGAGATCTGATTACGTTGGTGAAACTTACACAAGAAGATTAGATAAATTGAAGAGAGATGTTAAGCCAATGTTGGGTAAAGTTAAGAAACCATTGGATCAATTGGAATTAATCGATGTTTTGCAAAGATTGGGTATCTATTACCATTTCAAGGATGAAATTAAAAGAATTTTAAATTCTATCTATAATCAATACAATAGACATGAAGAATGGCAAAAAGATGATTTGTATGCTACTGCTTTGGAGTTTAGATTGTTAAGACAACATGGTTACGATGTTCCACAAGATGTTTTTAGTAGATTCAAAGATGATACAGGTTCTTTTAAAGCTTGTTTGTGTGAAGATATGAAGGGCATGTTGTGTTTGTACGAAGCATCATACTTGTGTGTTCAGGGTGAATCTACTATGGAACAAGCTAGAGATTTTGCACATAGACATTTGGGTAAAGGTTTGGAACAAAACATCGATCAAAATTTGGCTATCGAAGTTAAGCATGCATTGGAATTACCATTGCATTGGAGAATGCCAAGATTGGAAGCTAGATGGTTCATCGATGTTTACGAAAAGAGACAAGATATGAACCCAATCTTGTTAGAATTTGCTAAGTTGGATTTTAATATGGTTCAAGCAACTCATCAAGAAGATTTGAGACATATGTCATCTTGGTGGTCATCTACAAGATTGGGTGAAAAGTTGAACTTCGCTAGAGATAGATTGATGGAAAATTTCTTGTGGACTGTTGGTGTTATTTTCGAACCACAATACGGTTACTGTAGAAGAATGTCTACAAAGGTTAACACTTTGATCACAATCATTGATGATGTTTATGATGTTTACGGTACTATGGATGAATTAGAATTGTTTACAGATGTTGTTGATAGATGGGATATTAATGCTATGGACCCATTGCCAGAATACATGAAGTTGTGTTTCTTGGCATTGTACAACTCAACAAACGAAATGGCTTACGATGCATTGAAGGAACATGGTTTGCATATCGTTTCTTATTTGAGAAAGGCTTGGTCAGATTTGTGTAAGTCTTACTTGTTAGAAGCAAAGTGGTACTACTCAAGATACACTCCATCTTTGCAAGAATACATCTCAAATTCTTGGATCTCAATCTCTGGTCCAGTTATTTTGGTTCATGCTTACTTTTTGGTTGCAAACCCAATCACAAAGGAAGCTTTGCAATCATTGGAAAGATACCATAACATCATCAGATGGTCATCTATGATCTTGAGATTGTCAGATGATTTGGGTACTTCTTTAGATGAATTGAAGAGAGGTGACGTTCCAAAGTCTATCCAATGTTACATGTACGAAACAGGTGCTTCAGAAGAAGATGCAAGAAAGCATACTTCTTATTTGATCGGTGAAACATGGAAGAAATTGAATGAAGATGGTGCTGTTGAATCACCATTCCCAGAAACTTTTATTGGTATCGCAATGAATTTGGCTAGAATGGCACAATGTATGTATCAACATGGTGACGGTCATGGTATTGAATACGGTGAAACAGAAGATAGAGTTTTGTCTTTGTTGGTTGAACCAATCCCATCATTGTCTTTTGAATAA |
| --- |

The predicted chloroplast targeting peptide is marked in red bold font.
